# Elemental Analysis of Aging Human Pituitary Glands Implicates Mercury as a Contributor to the Somatopause

**DOI:** 10.3389/fendo.2019.00419

**Published:** 2019-06-26

**Authors:** Roger Pamphlett, Stephen Kum Jew, Philip A. Doble, David P. Bishop

**Affiliations:** ^1^Discipline of Pathology, Brain and Mind Centre, Sydney Medical School, The University of Sydney, Sydney, NSW, Australia; ^2^Department of Neuropathology, Royal Prince Alfred Hospital, Sydney, NSW, Australia; ^3^The Atomic Medicine Initiative, University of Technology, Sydney, NSW, Australia

**Keywords:** mercury, pituitary gland, aging, growth hormone, somatopause, somatotroph

## Abstract

**Background:** Growth hormone levels often decline on aging, and this “somatopause” is associated with muscle and bone loss, visceral adiposity and impaired cardiovascular function. Mercury has been detected in human pituitary glands, so to see if mercury could play a part in the somatopause we measured the proportion of people at different ages who had mercury in their anterior pituitary cells.

**Materials and methods:** Paraffin sections of pituitary glands taken at autopsy from 94 people between the ages of 2 and 99 years were stained for inorganic mercury using autometallography. Pituitary mercury content was classified as none, low (<30% of cells) or high (>30% of cells) in increasing two-decade age groups. Autometallography combined with immunohistochemistry determined which hormone-producing cells contained mercury. Laser ablation-inductively coupled plasma-mass spectrometry was used to confirm the presence of mercury.

**Results:** The proportion of people with low-content pituitary mercury remained between 33 and 42% at all ages. The proportion of people with high-content mercury increased with increasing age, from 0% of people in the 2–20 year group to a peak of 50% of people in the 61–80 years group, followed by a fall to 35% of people in the 81–99 years group. Mercury, when present, was found always in somatotrophs, occasionally in corticotrophs, rarely in thyrotrophs and gonadotrophs, and never in lactotrophs. Laser ablation-inductively coupled plasma-mass spectrometry detected mercury in regions of pituitaries that stained with autometallography.

**Conclusions:** The proportion of people with mercury in their anterior pituitary cells, mostly somatotrophs, increases with aging, suggesting that mercury toxicity could be one factor contributing to the decline in growth hormone levels found in advancing age.

## Introduction

In many people growth hormone levels decline with increasing age, a phenomenon termed the somatopause (1–8). Although average growth hormone levels decrease on aging, values for individuals at the same ages vary, so that 45% of people aged over 69 years still release growth hormone normally (3). Obesity plays some role in the somatopause, but most factors behind the variability of growth hormone levels in aging remain unknown (3). The somatopause is linked to several conditions associated with aging, such as decreases in muscle mass, bone density, and skin thickness, as well as cognitive and cardiovascular dysfunction, frailty and falls, and visceral adiposity (9, 10). Growth hormone replacement in older men ameliorates some of these changes (4), but the value of growth hormone replacement in the somatopause remains controversial (11–15). Finding some of the factors underlying the somatopause could, however, be of value in developing ways to prevent or treat declining levels of growth hormone.

The rodent anterior pituitary readily takes up organic mercury, which localizes to lysosomes and secretory granules of somatotrophs (16). The primate anterior pituitary takes up mercury vapor from exposure to as few as three dental mercury-containing amalgam fillings, with the mercury again seen in lysosomes and secretory granules of somatotrophs (17). In humans, the anterior pituitary is a preferred organ for mercury deposition (18), since mercury has been found in the anterior pituitary by several workers (19–23), but which hormone-producing pituitary cells contained the mercury was not able to be established in these studies. Pituitary mercury levels correlate with numbers of dental amalgam fillings (24–27), but the small numbers and limited age ranges of people studied here did not allow for age-related analyses.

To determine whether age-related accumulation of mercury within the pituitary could play a part in causing the somatopause, we examined pituitary glands from people over a wide range of ages, using a histochemical technique that detects intracellular mercury, and combined this with hormone immunohistochemistry to see if human somatotrophs preferentially take up mercury.

## Materials and Methods

### Study Design

Autopsy reports between 1997 and 2014 from the New South Wales Department of Forensic Medicine were filtered to include a variety of clinicopathological conditions, such as cancer, neurodegenerative diseases, and psychiatric disorders, as well as people who died from sudden non-medical causes of death such as drowning, suicide or trauma. Horizontally-sectioned pituitary samples taken from 94 of these individuals (62 male and 32 female) (Table 1) were studied.

**Table 1:** Ages, genders, major diagnoses, and causes of death in people with no, low, or high-content pituitary mercury.

**Age (year)**	**Gender**	**Clinicopathology**	**Cause of death**	**Pituitary mercury content**
2–20	Male	None	Drowning	None
2–20	Male	None	Drowning	None
2–20	Male	None	Drug OD	None
2–20	Female	None	Trauma	None
2–20	Female	None	Trauma	None
2–20	Male	None	Drowning	Low
2–20	Male	Depression	Suicide	Low
2–20	Male	None	Suicide	Low
2–20	Male	None	Suicide	None
21–40	Female	None	Suicide	High
21–40	Male	None	Suicide	High
21–40	Male	None	Suicide	High
21–40	Male	None	Cardiovascular	None
21–40	Male	None	Drowning	None
21–40	Male	Schizophrenia	Drug OD	None
21–40	Male	Schizophrenia	Drug OD	None
21–40	Female	Epilepsy	SUDEP	None
21–40	Male	Schizophrenia	Unknown	None
21–40	Male	Obesity	Cerebrovascular	Low
21–40	Female	None	Drowning	Low
21–40	Male	None	Drowning	Low
21–40	Male	None	Drowning	Low
21–40	Male	None	Drug OD	Low
21–40	Male	None	Suicide	Low
21–40	Male	Depression	Suicide	Low
21–40	Female	Schizophrenia	Suicide	Low
21–40	Female	Anorexia nervosa	Undernutrition	Low
21–40	Female	None	Drowning	High
21–40	Male	Schizophrenia	SUDEP	High
21–40	Male	Schizophrenia	Suicide	Low
21–40	Male	None	Suicide	Low
21–40	Male	None	Suicide	None
21–40	Male	None	Suicide	None
21–40	Male	None	Suicide	None
21–40	Male	None	Suicide	None
41–60	Male	None	Suicide	High
41–60	Male	None	Suicide	High
41–60	Female	None	Suicide	High
41–60	Male	None	Suicide	High
41–60	Male	None	Suicide	Low
41–60	Male	Obesity	Cardiovascular	None
41–60	Male	Schizophrenia	Drug OD	None
41–60	Male	Schizophrenia	Drug OD	None
41–60	Male	Schizophrenia	Respiratory	None
41–60	Female	Schizophrenia	Suicide	Low
41–60	Male	Bipolar	Trauma	None
41–60	Male	None	Cancer	Low
41–60	Female	Schizophrenia	Cirrhosis	Low
41–60	Male	None	Drowning	Low
41–60	Male	None	Drug OD	Low
41–60	Male	Schizophrenia	Drug OD	Low
41–60	Male	None	Trauma	Low
41–60	Female	None	Trauma	Low
41–60	Male	Schizophrenia	Unknown	Low
41–60	Female	None	Drowning	High
41–60	Female	None	Drowning	High
41–60	Female	Bipolar	Drug OD	High
41–60	Male	Obesity	Drug OD	High
41–60	Male	MSA	Unknown	High
41–60	Male	None	Suicide	None
41–60	Male	None	Suicide	None
61–80	Male	Depression	Suicide	Low
61–80	Male	None	Suicide	Low
61–80	Male	Alzheimer	Drowning	None
61–80	Female	Alzheimer	Infection	None
61–80	Female	Parkinson	Asphyxia	Low
61–80	Male	Parkinson	Cardiovascular	Low
61–80	Male	MSA	Choking	Low
61–80	Male	Obesity	Trauma	Low
61–80	Female	Parkinson	Cardiovascular	High
61–80	Male	MSA	Choking	High
61–80	Female	None	Drowning	High
61–80	Male	None	Drowning	High
61–80	Female	Parkinson	Infection	High
61–80	Female	Parkinson	Trauma	High
61–80	Female	None	Trauma	High
61–80	Male	None	Trauma	High
81–99	Female	None	Cardiovascular	None
81–99	Female	Alzheimer	Cardiovascular	None
81–99	Male	Dementia NOS	Cardiovascular	None
81–99	Female	Lewy body disease	Infection	None
81–99	Male	None	Trauma	None
81–99	Female	None	Cardiovascular	Low
81–99	Female	None	Cardiovascular	Low
81–99	Male	None	Cardiovascular	Low
81–99	Female	Alzheimer	Cardiovascular	Low
81–99	Male	Parkinson	Infection	Low
81–99	Male	Alzheimer	Trauma	Low
81–99	Female	None	Cardiovascular	High
81–99	Female	Parkinson	Cardiovascular	High
81–99	Male	PSP	Cardiovascular	High
81–99	Male	Lewy body disease	Cerebrovascular	High
81–99	Female	Alzheimer	Infection	High
81–99	Female	Alzheimer	Infection	High

*OD, overdose; MSA, multiple system atrophy; NOS, not otherwise specified; PSP, progressive supranuclear palsy; SUDEP, sudden unexpected death in epilepsy*.

### Ethics

The study was approved by the Human Research Committee, Sydney Local Health District RPAH Zone, and by the Office of the New South Wales Coroner. The institutional review board waived the need for written informed consent from relatives of individuals studied since this was a de-identified retrospective study of autopsy tissue.

### Autometallography

**Figure 1:**
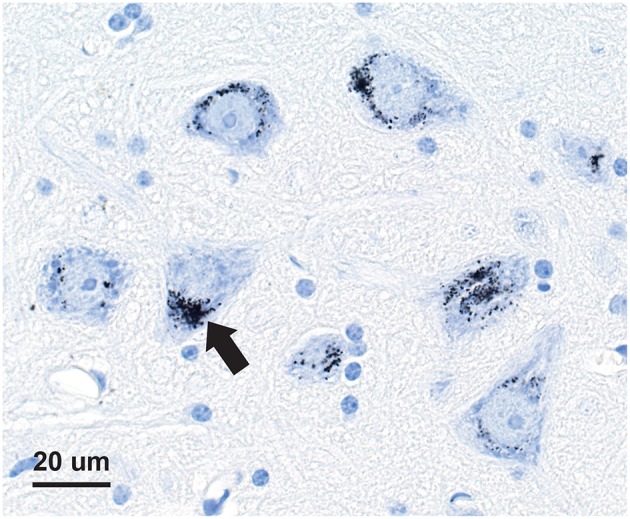
Mercury-positive control section for autometallography. In a mouse given intraperitoneal mercuric chloride, mercury is seen as multiple black granules (e.g., arrow) in the cytoplasm of spinal anterior horn motor neurons. Autometallography/hematoxylin.

Seven-micron paraffin sections of the pituitary were stained for inorganic mercury bound to sulfide or selenide using silver nitrate autometallography, which represents the presence of mercury as black grains (28). Autometallography is a sensitive photographic technique that can detect as few as 10 mercury sulfide/selenide molecules in a cell (29). Briefly, sections were placed in physical developer containing 50% gum arabic, citrate buffer, hydroquinone, and silver nitrate at 26°C for 80 min in the dark then washed in 5% sodium thiosulphate to remove unbound silver. Sections were counterstained with mercury-free hematoxylin and viewed with bright-field microscopy. Each staining run included a control section of mouse spinal cord where motor neuron cell bodies contained mercury (Figure 1) following an intraperitoneal injection of mercuric chloride (30). Sections were also stained with hematoxylin only to act as a control, and hematoxylin and eosin to assess histopathology. Of note, the semi-quantitative use of autometallography (modified to detect zinc) has been used previously in relation to endocrine studies of insulin and glucose levels (31).

### Mercury Content of the Pituitary

The proportion of pituitary cells containing mercury was estimated by counting the number of cells containing mercury (defined as cells with two or more cytoplasmic autometallography grains) within a 10 × 10 eyepiece grid viewed at a magnification of 200× , covering an area of 1 × 1 mm, which was stepped through the pituitary. Mercury content within the anterior pituitary was categorized as being “none” if no mercury-containing cells were seen, “low” if mercury was present in fewer than 30% of cells throughout the pituitary, and “high” if, in at least four regions, mercury was present in more than 30% of cells.

### Pituitary Hormone Immunostaining

To find which particular hormone-producing pituitary cells contained mercury, after autometallography staining 11 pituitaries known to contain mercury were immunostained with mouse anti-human antibodies to growth hormone 1:2,000 (polyclonal, Dako), adrenocorticotropic hormone 1:250 (clone 02A3, Dako), thyroid stimulating hormone 1:100 (clone 42, Dako), prolactin 1:500 (polyclonal, Cell Marque), luteinising hormone 1:20,000 (polyclonal, Dako), and follicle stimulating hormone 1:200 (clone C10, Dako). Visualization was with Bond Polymer Refine Red Detection (Leica) so as not to obscure the black mercury grains. The proportion of each type of hormone-containing pituitary cell that contained mercury was graded into four categories as: none (0%), rare (1–5%), occasional (6–30%), and common (>30%).

### Laser Ablation-Inductively Coupled Plasma-Mass Spectrometry (LA-ICP-MS)

In addition to inorganic mercury, autometallography can stain inorganic silver, bismuth, and gold (28, 32). Therefore, to confirm that autometallography staining in pituitary cells was due to mercury, seven-micron paraffin sections of selected pituitaries, in which regional differences in autometallography staining were marked, were deparaffinised and subjected to LA-ICP-MS for mercury, silver, bismuth, and gold. In two pituitaries LA-ICP-MS was used to look for aluminum, cadmium and lead, as well as the above metals. An NWR193 excimer laser (Kenelec Scientific) was hyphenated to an Agilent Technologies 7700 ICP-MS fitted with “s” lenses for enhanced sensitivity, with argon used as the carrier gas. LA-ICP-MS conditions were optimized on NIST 612 Trace Element in Glass CRM and the sample was ablated with a 55 μm spot size and a scan speed of 220 μm s^−1^ at a frequency of 20 Hz. The data were collated into a single image file using in-house developed software and visualized using FIJI. Limits of detection using LA-ICP-MS were estimated to be between 0.05 and 0.81 μg g^−1^ (33).

### Statistics

GraphPad Prism 8 software was used for chi-square tests for trend to analyse the proportion of people with any mercury in their pituitary in relation to age. Significance was assessed at the 0.05 level.

## Results

### Mercury in Pituitary Cells

**Figure 2:**
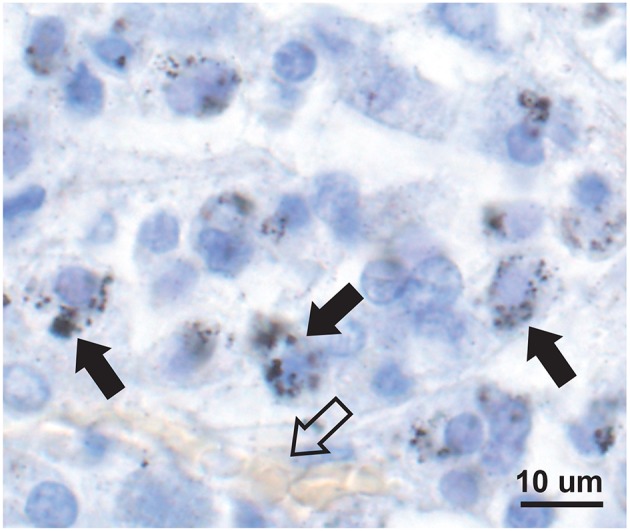
Mercury in anterior pituitary cells. Mercury is seen as multiple small black autometallography-stained foci of varying size within the pale cytoplasm of cells (e.g., solid arrows), surrounding the blue-stained nuclei. A small blood vessel with pale yellow erythrocytes is present (open arrow). Autometallography/hematoxylin.

**Figure 3:**
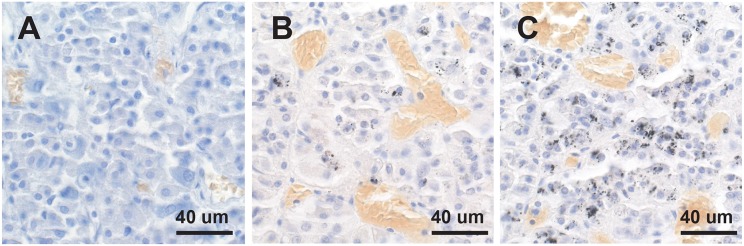
No, low or high mercury content in the anterior pituitary. **(A)** No mercury (i.e., no autometallography staining) is present in this pituitary. **(B)** Low mercury content (autometallography grains in fewer than 30% of cells) of the pituitary is seen in this pituitary. **(C)** High mercury content (more than 30% of cells containing autometallography grains) is present in this pituitary. Numerous small blood vessels (with yellow-colored erythrocytes) are present in all pituitaries. Autometallography/hematoxylin.

Mercury-positive autometallography staining of pituitary cells was seen as multiple small round black grains of varying size within the cytoplasm (Figure 2). The distribution of mercury-containing cells within the pituitary could be widespread or regional, often with preference for the lateral lobes. The percentage of pituitary samples in each mercury-content category (Figure 3) was 32% for no mercury, 38% for low mercury, and 30% for high mercury. No autometallography staining was seen in the posterior pituitary, no black grains were seen in sections stained with hematoxylin alone, and no incidental pathology was present in any of the pituitary samples.

### Proportion of People at Different Ages With Mercury in Their Pituitary Glands

**Figure 4:**
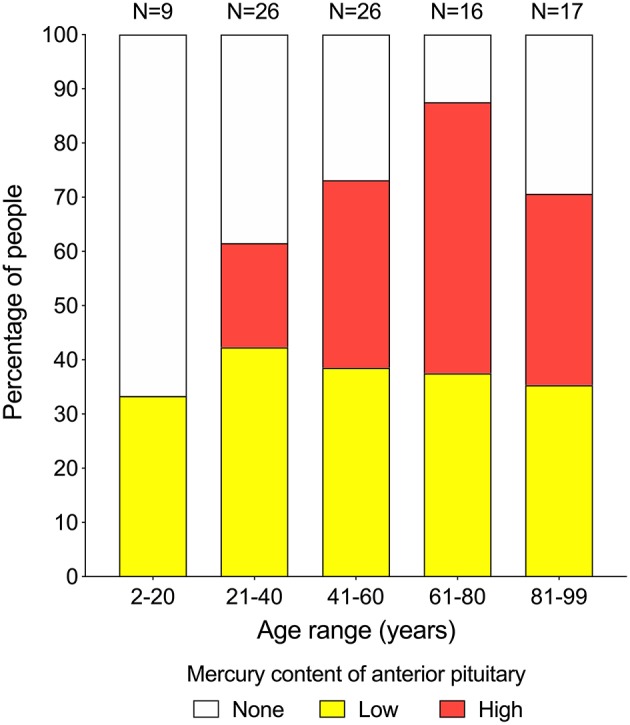
Proportion of people at increasing ages with pituitaries containing mercury. Low-content mercury (yellow bars) is present in similar proportions of people (between 33 and 42%) over all age ranges. High-content mercury (red bars) starts appearing in the 21–40 years group, then increases with advancing age to reach a peak of 50% of people in the 61–80 years group, before dropping back in the final 81–99 years group. Numbers of people in each group are given above the bars.

Mercury (either low or high content) was seen in the anterior pituitary in 33% of people in the 2–20 years group (*N* = 9), 62% in the 21–40 years group (*N* = 26), 73% in the 41–60 years group (*N* = 26), 88% in the 61–80 years group (*N* = 16), and 71% of people in the 81–99 years group (*N* = 17) (Figure 3). The proportion of people with low-content pituitary mercury remained between 33 and 42% at all ages. The proportion of people with high-content mercury increased with increasing age, from 0% of people in the 2–20 years group to a peak of 50% of people in the 61–80 years group, followed by a fall to 35% of people in the 81–99 years group (Figure 4). The overall increase in the proportion of people on aging who had any mercury in their pituitaries was significant (chi-square trend for aging 4.5, *p* = 0.034). The youngest person to have pituitary mercury was aged 16 years.

The 32 females in the study had a greater proportion (78%) of mercury in their pituitaries than the 62 males (55%), probably because females were older on average (mean age 63 years, age range 2–99 years, SD 28 years) than males (mean age 47 years, age range 2–96 years, SD 23 years). The numbers of people with mercury in their pituitary cells did not appear to be markedly higher for any individual clinicopathological condition or cause of death (Table 1), though numbers in each diagnostic group within two-decade age ranges were too small for statistical comparisons.

### Combined Autometallography and Pituitary Hormone Immunostaining

**Figure 5:**
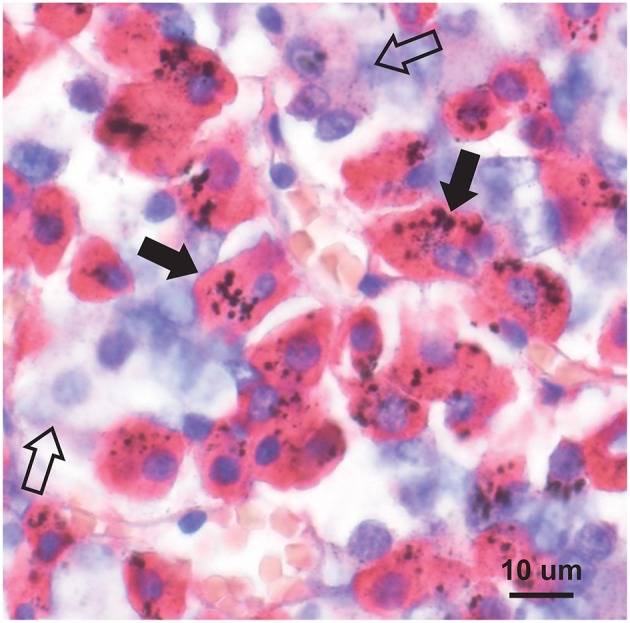
Mercury in pituitary immunostained somatotrophs. Mercury is seen as clusters of round black grains in the red-immunostained cytoplasm of somatotrophs (e.g., closed arrows). Cells not containing growth hormone (e.g., open arrows) do not contain mercury. Autometallography/hematoxylin combined with growth hormone immunostaining.

The usual geographic predominance of the different pituitary hormone-containing cells was found in the 11 pituitaries studied with immunostaining, i.e., somatotrophs in the lateral lobes, corticotrophs in the central area, thyrotrophs in the central anterior area, lactotrophs in the posterolateral areas, and gonadotrophs widely dispersed. In the eight people with high-content pituitary mercury, the mercury was seen in many growth hormone-immunostained somatotrophs (Figure 5), in occasional immunostained corticotrophs and thyrotrophs, in rare immunostained gonadotrophs, but not in immunostained lactotrophs (Table 2). In the three people with low-content pituitary mercury, the mercury was seen in occasional somatotrophs, in rare corticotrophs, but not in thyrotrophs, gonadotrophs, or lactotrophs. The intensity of immunostaining was similar in cells with and without mercury, indicating that the autometallography process itself did not interfere with the immunostaining.

**Table 2:** Proportion of pituitary hormone-immunostained cells containing mercury.

**Age (year)**	**Pituitary mercury content**	**GH**	**ACTH**	**TSH**	**LH**	**FSH**	**PROL**
30	High	+++	+	+	+	0	0
44	High	+++	+	0	+	+	0
46	High	+++	+	+	0	0	0
48	High	+++	++	+	0	0	0
49	High	+++	+	0	0	0	0
59	High	+++	+	0	0	0	0
72	High	+++	++	+	+	0	0
95	High	+++	++	0	0	0	0
29	Low	++	0	0	0	0	0
36	Low	++	+	0	0	0	0
38	Low	++	0	0	0	0	0

*0, 0% (none); +, 1–5% (rare); ++, 6–30% (occasional); +++, >30% (common)*.

### Correlation of Autometallography With Laser Ablation-Inductively Coupled Plasma-Mass Spectrometry (LA-ICP-MS)

**Figure 6:**
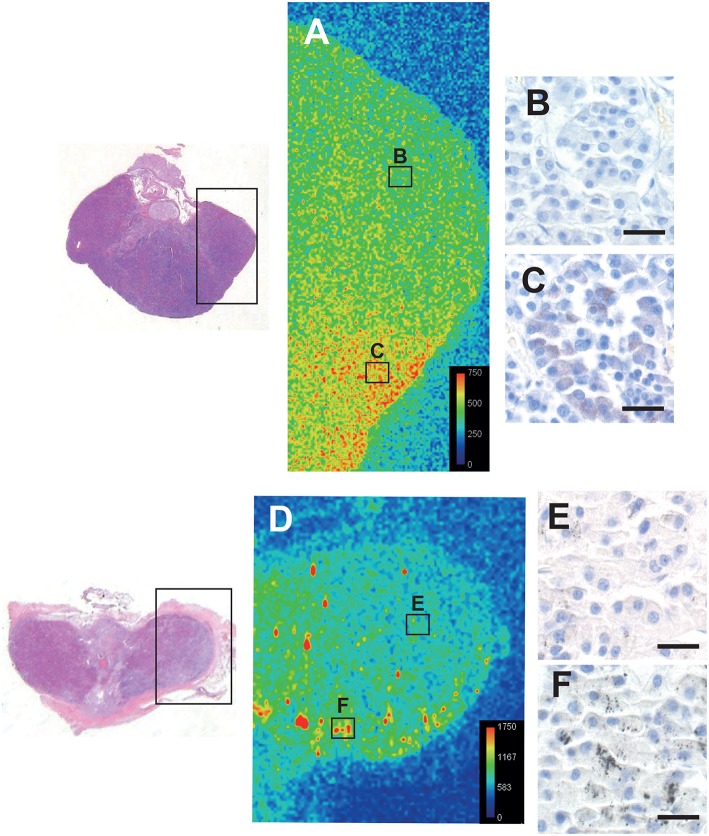
Correlation of autometallography with LA-ICP-MS. The regions of two pituitaries scanned with LA-ICP-MS are within the boxes on the left. **(A)** LA-ICP-MS image from a 16-years old male shows minimal mercury (green) on the upper right (B box region), correlating with **(B)** negative autometallography staining for mercury in this region. LA-ICP-MS high mercury levels (red and yellow) are present on the lower right of this pituitary (C box region) (LA-ICP-MS intensity scale in counts per second), correlating with **(C)** finely-dispersed autometallography staining here. **(D)** LA-ICP-MS image from a 75 years-old male shows minimal mercury (green/blue) on the upper right (E box region), corresponding to **(E)** pituitary cells containing sparse autometallography grains here. Large clusters of LA-ICP-MS mercury (yellow/red) are scattered within the left and lower regions of this pituitary (e.g., F box region), corresponding to **(F)** the dense autometallography grains in pituitary cells here. Note the higher numbers of counts per second for each color level in this pituitary. Hematoxylin and eosin on the two sections on the left. Autometallography/hematoxylin on B, C, E, and F, bars = 20 μm.

LA-ICP-MS of the pituitaries of a 16 years-old male with low-content autometallography mercury, and a 75 years-old male and a 44 years-old female both with high-content autometallography mercury, confirmed that autometallography staining in different regions of the pituitary was due to the presence of mercury (Figure 6). No significant amounts of the other metals that can be detected with autometallography, i.e., inorganic silver, bismuth, and gold, were seen on LA-ICP-MS. No LA-ICP-MS mercury was found in a 78 years-old woman with no autometallography staining of the pituitary. No significant accumulation of pituitary aluminum, cadmium, or lead was seen.

## Discussion

Key findings of this study were that mercury accumulates over time in human pituitary cells, and that the cells that predominantly contain mercury are the growth hormone-containing somatotrophs. Up to fifty percent of older adults have pituitaries with a high content of mercury, which is approximately the same proportion of older adults who have reduced secretion of growth hormone (3). These findings indicate that mercury is a candidate for an environmental toxin that causes the reduced growth hormone levels of the somatopause.

The major physical forms of mercury are mercury vapor, methylmercury, and mercuric ions, the latter being the proximate toxic intracellular form of the metal (34). Exposure to all these three forms of mercury results in detectable levels of mercury in the pituitary (16, 27, 35). Humans are usually exposed to mercury either as methylmercury from eating mercury-containing fish, or mercury vapor from occupational exposures or dental amalgam restorations.

The reason somatotrophs preferentially take up mercury is not clear. The pituitary may be predisposed to taking up circulating toxins such as mercury because of its rich blood supply, and because, unlike the brain, it does not have endothelial tight junctions to prevent the entry of these metals. Mercury vapor crosses cell membranes readily and is oxidized to ionic mercury by the intracellular catalase-hydrogen peroxide pathway, so that cells rich in these enzymes are more likely to trap mercuric ions within the cell (34). Methylmercury crosses cell membranes and is then slowly demethylated in the cell into mercuric ions. Mercury is transported actively into cells of the kidney, liver, intestine, and placenta (36), and somatotrophs may have similar as-yet unknown active transport mechanisms for mercury. Corticotrophs, thyrotrophs, and gonadotrophs in our study that occasionally contained mercury are cells known to co-express growth hormone (37), so the presence of some growth hormone in a cell may be a clue that it is susceptible to the entry of mercury.

Mercury damages cells via the generation of oxygen radicals, binding to membranous organelles such as mitochondria, binding to sulfhydryl-containing proteins, promoting autoimmunity, and causing genetic or epigenetic changes (18, 38). The extensive immunostaining of growth hormone in cells that contained large amount of mercury indicate that growth hormone production in these somatotrophs appears intact, so a deleterious effect of mercury on growth hormone secretion, rather than production, appears likely. Lysosomes store intracellular toxicants such as mercury, and mercury is located within lysosomes in the pituitary cells of rats exposed to mercury as seen on electron microscopy (16, 35, 39). The distribution of mercury in our human pituitary cells suggests that mercury is also within lysosomes, though this could not be confirmed using electron microscopy because of the method of tissue processing. Mercury accumulation in rodent somatotrophs causes vacuolation of lysosomes, indicating disruption to their structure (16). A build-up of mercury within lysosomes could disturb their many functions, which, as well as autophagy, include cell signaling and metabolic roles (40). Although lysosomal damage seems a likely cause of decreased growth hormone secretion in mercury-containing somatotrophs, mercury also binds to pituitary cell secretory granules (16, 35, 39), another way in which mercury could inhibit growth hormone secretion.

The physiological effects of mercury on human growth hormone secretion is not yet known. A variety of pituitary hormones have been measured in different groups of people occupationally exposed to mercury (41), but none of these included growth hormone levels, probably because of the challenges in analyzing the diurnal and nocturnal fluctuations in growth hormone secretion (3). Analyses of growth hormone secretion in experimental animals exposed to mercury may cast light on this issue.

Growth hormone causes an increase in lean body mass and a decrease in adipose mass, and visceral adiposity is a key feature of the somatopause (42). The amount of visceral adipose tissue is associated with blood mercury concentration (43), but the reason for this is not known. Our findings suggest the link could be mercury in somatotrophs decreasing the secretion of growth hormone, with consequent re-partitioning of adipose tissue to the visceral compartment.

The increase in the proportion of people with high-content pituitary mercury peaked in the 61–80 years group, but then fell back in the oldest 81–99 years group. We found a similar decrease in the proportion of people who had toxic metals in their locus ceruleus in the oldest age group (44). These findings could be due to a “survivor” effect, in which people who live to advanced ages are less likely to have been exposed to high levels of environmental toxins, with consequent deleterious effects on health. This survivor effect has been suggested to be a reason for the reduced incidence in advanced age of Alzheimer disease, Parkinson disease, and amyotrophic lateral sclerosis, disorders that have been associated with exposure to toxic metals, as well as for the plateau of mortality in later life (44).

This study has several limitations (1) This was a retrospective study of pituitaries sampled at coronial autopsy, so we do not have details of possible mercury exposure during life, e.g., frequency and type of fish consumption, numbers of occlusal amalgam restorations, and lifetime occupations. (2) It is difficult to obtain enough human pituitary autopsy samples, together with detailed clinical data and biochemical analyses of growth hormone secretion, to allow a comprehensive examination of the effects of mercury on growth hormone levels throughout life. Ideally, an ambulant population in whom pituitary mercury could be estimated by a future non-invasive technique, and in whom growth hormone levels could be measured, would be studied. (3) Pituitaries were not sampled at the same horizontal level, and mercury distribution in pituitaries was often patchy, so no formal quantification of total numbers of pituitary cells containing mercury was attempted. (4) LA-ICP-MS was unable to be undertaken on all pituitaries, so we cannot say with certainty that autometallography demonstrated mercury in all individuals. However, a previous study using LA-ICP-MS indicated that autometallography in human tissue most likely shows the presence of mercury (44). (5) The youngest age at which pituitary mercury was found in our study was 16 years, but we were not able to accurately determine at what age pituitary mercury first starts to appear, since numbers in the 2–20 years group were relatively small, and the three people with pituitary mercury were all in the second decade of life. (6) Autometallography demonstrates inorganic mercury that is bound to sulfides and selenides, but not organic mercury. However, inorganic mercury is considered to be the proximate toxic form of the metal in tissues (34) and is therefore the most important type to measure. (7) Post mortem intervals are seldom able to be determined accurately in coronial autopsies. We did not therefore attempt to detect types of cell damage or death, such as apoptosis or free radical generation, that have been described in mercury toxicity. However, these mechanisms could be sought in rodent models since mercury accumulation in the rat pituitary is well described (16).

In conclusion, we propose that mercury in pituitary somatotrophs plays a part in the age-related decline of growth hormone that is found in many people. The amount of mercury in the atmosphere is increasing steadily, due mostly to the burning of fossil fuels (45), and this mercury enters the global atmosphere-water-soil cycle. Therefore, if mercury exposure does cause decreased growth hormone secretion, the numbers of people suffering from effects of the somatopause can be expected to increase in future years. Although there is as yet no unequivocal link between mercury in the somatopause, it would seem prudent to limit consumption of high-mercury predatory fish such as shark and swordfish, maintain strict workplace protections against mercury exposure, consider alternatives to the placement of mercury-containing amalgam fillings, and ensure that the diet contains enough selenium to help counteract any toxic effects of mercury (46).

## Data Availability

All datasets generated for this study are included in the manuscript and/or the supplementary files.

## Ethics Statement

The study was approved by the Human Research Committee, Sydney Local Health District RPAH Zone, and by the Office of the New South Wales Coroner. The institutional review board waived the need for written informed consent from relatives of individuals studied since this was a de-identified retrospective study of autopsy tissue.

## Author Contributions

RP conceived and planned the study and drafted the manuscript. RP, SK, DB, and PD performed the experiments and analyzed the data. All authors revised and approved the article.

### Conflict of Interest Statement

The authors declare that the research was conducted in the absence of any commercial or financial relationships that could be construed as a potential conflict of interest.
